# Contemporary genetic structure affects genetic stock identification of steelhead trout in the Snake River basin

**DOI:** 10.1002/ece3.6708

**Published:** 2020-08-28

**Authors:** John H. Powell, Matthew R. Campbell

**Affiliations:** ^1^ Idaho Department of Fish and Game Eagle Idaho USA

**Keywords:** genetic stock identification, genetic structure, isolation by distance, steelhead trout

## Abstract

Genetic stock identification is a widely applied tool for the mixed‐stock management of salmonid species throughout the North Pacific Rim. The effectiveness of genetic stock identification is dependent on the level of differentiation among stocks which is often high due to the life history of these species that involves high homing fidelity to their natal streams. However, the utility of this tool can be reduced when natural genetic structuring has been altered by hatchery translocation and/or supplementation. We examined the genetic population structure of ESA‐listed steelhead in the Snake River basin of the United States. We analyzed 9,613 natural‐origin adult steelhead returning to Passive Integrated Transponder detection sites throughout the basin from 2010 through 2017. Individuals were genotyped at 180 single nucleotide polymorphic genetic markers and grouped into 20 populations based on their return location. While we expected to observe a common pattern of hierarchical genetic structuring due to isolation by distance, we observed low genetic differentiation between populations in the upper Salmon River basin compared to geographically distant populations in the lower Snake River basin. These results were consistent with lower genetic stock assignment probabilities observed for populations in this upper basin. We attribute these patterns of reduced genetic structure to the translocation of lower basin steelhead stocks and ongoing hatchery programs in the upper Salmon River basin. We discuss the implications of these findings on the utility of genetic stock identification in the basin and discuss opportunities for increasing assignment probabilities in the face of low genetic structure.

## Graphical abstract

The genetic population structure of ESA‐listed steelhead in the Snake River basin of the United States was examined using 9,613 natural‐origin adult steelhead returning to Passive Integrated Transponder detection sites from 2010 through 2017. We observed low genetic differentiation between populations in the upper Salmon River basin and geographically distant populations in the lower Snake River basin consistent with lower genetic stock assignment probabilities observed for populations in this upper basin. We attribute these patterns of reduced genetic structure to the translocation of lower basin steelhead stocks and ongoing hatchery programs in the upper Salmon River basin and discuss the implications of these findings on the implementation of genetic stock identification in the basin.

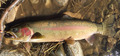

## 1 INTRODUCTION

**FIGURE 1:**
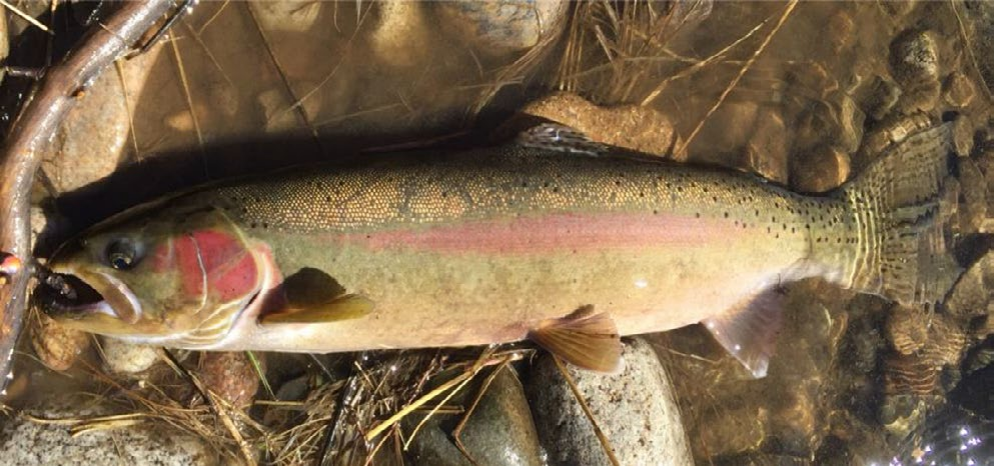
Adult steelhead from the upper Salmon River. Photo credit: Matthew R. Campbell

Genetic stock identification (GSI) is a widely used tool for managing fish populations throughout the North Pacific Rim (e.g., Beacham et al., 2006, 2017; Beacham, Spilsted, Le, & Wetklo, 2008; Dann, Habicht, Baker, & Seeb, 2013; Hess et al., 2016; Hess, Campbell, Matala, & Narum, 2014; Hess, Whiteaker, Fryer, & Narum, 2014). For example, conducting GSI on sockeye salmon (*Oncorhynchus nerka*) caught in the Port Moller test fishery allowed real‐time shifts in fishing effort in Bristol Bay to reduce the risk of overharvesting low abundance stocks in the mixed‐stock fishery (Dann et al., 2013). In addition, GSI has been used to monitor the status and trends of steelhead (*O. mykiss*, Figure 1) stocks in the Snake River basin that are listed as threatened under the US Endangered Species Act (Northwest Fisheries Science Center, 2015). This method makes use of genetic data from reference populations (representing the contributing stocks) as a baseline to assign fish of unknown origin (e.g., Anderson, Waples, & Kalinowski, 2008; Hasselman et al., 2016; Shaklee, Beacham, Seeb, & White, 1999). Genetic stock identification is most effective when species are phylopatric, and restricted dispersal among populations leads to significant levels of genetic differentiation (Araujo, Candy, Beacham, White, & Wallace, 2014). Many salmonid species exhibit both strong homing fidelity and genetic structuring making GSI an effective tool to use in their management.

Despite its widespread use, the accuracy of GSI can be reduced when natural genetic structuring has been altered by hatchery translocation and/or supplementation with out‐of‐basin stocks. For example, Pearse, Martinez, and Garza (2011), found that anthropogenic changes to wild coastal steelhead populations in California, including hatchery supplementation with common stocks, reduced genetic differentiation relative to historic conditions. Reductions in genetic differentiation among populations can adversely affect the accuracy of genetic stock identification. Substantial error can occur in estimating stock compositions when mean *F*_ST_ among populations is less than 0.01 (Araujo et al., 2014). Because hatchery supplementation programs are widespread throughout the North Pacific Rim understanding changes to genetic structure across the landscape from these programs is an important step in accounting for uncertainty in GSI analyses.

Throughout the Columbia River basin of Washington, Oregon, and Idaho, GSI is widely used to inform management of steelhead for both harvest (e.g., Byrne et al., 2018) and to monitor status and trends for populations listed under the Endangered Species Act (Northwest Fisheries Science Center, 2015). Matala, Ackerman, Campbell, and Narum (2014) found significant isolation by distance (IBD) in both coastal and inland steelhead lineages throughout the Columbia River basin. Isolation by distance would lead to the expectation that across basins the most genetically differentiated populations should occur in the streams located in the headwaters of the basins. However, patterns of IBD can be disrupted by anthropogenic influences, such as supplementation with non‐native stocks that alter dispersal across the landscape as was seen in California by Pearse et al. (2011).

**FIGURE 2:**
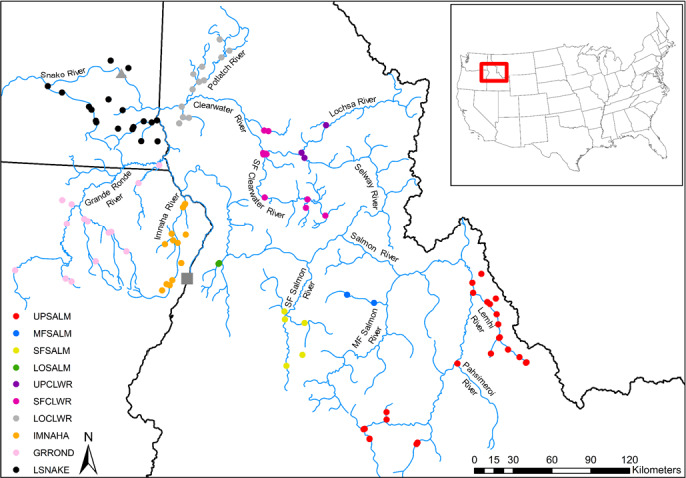
Location of adult steelhead sampling sites color coded by genetic stock. The gray triangle represents the Lower Granite Dam adult fish facility where returning steelhead were implanted with PIT tags and genetically sampled. Black circles represent PIT tag detection sites used in this study (Appendix). The gray square represents Hells Canyon Dam

Hatchery supplementation has been used in the Snake River, a tributary to the Columbia River, to mitigate for lost habitat and fisheries as a result of hydropower development (Busby et al., 1996). In addition, by the early 1960s, the Idaho Department of Fish and Game had initiated efforts to use captive‐reared fish to supplement or reestablish steelhead populations in their historically occupied range in Idaho (Bjornn, 1978). Beginning in 1966, efforts were made by the Idaho Department of Fish and Game to supplement steelhead in the upper Salmon River using fish trapped at the recently completed Hells Canyon Dam (Figure 2, Reingold, 1967). These translocations continued annually through 1972 and their success resulted in the founding of two hatchery populations in the upper Salmon River watershed (Stiefel, 2013). These hatchery populations serve as genetic repositories for the steelhead stocks that previously spawned above Hells Canyon Dam (where there is no fish passage), and provide harvest opportunities as part of legally mandated mitigation programs.

Previous research has documented historical steelhead translocation and supplementation efforts in the Snake River basin, but the consequences of such introductions on GSI have yet to be examined. The objective of this study was to describe the contemporary genetic structure of steelhead populations across the Snake River basin and assess the effects of historical translocations into the upper Salmon River watershed on patterns of IBD and GSI. To accomplish this objective, we genotyped representative samples from across the basin using fin tissues collected from natural‐origin adults at Lower Granite Dam whose last known locations were determined using Passive Integrated Transponder (PIT) tag detection sites. Lower Granite Dam provides a sampling point for all steelhead migrating upstream in the Snake River, and PIT tag arrays allow assignment of spawning locations for presumed natural‐origin fish. Returning adult steelhead with both a genetic sample and PIT tag detection can be used to assess the genetic structure in the basin. To assess the impacts of translocations on genetic structure we quantified patterns of IBD at a basin‐wide scale including and excluding supplemented populations in the upper Salmon River watershed. Finally, we assessed the accuracy and precision of GSI assignments of samples from the upper Salmon River watershed relative to other populations throughout the Snake River basin.

## 2 METHODS

### 2.1 GSI baseline

Vu et al. (2015) developed the Snake River steelhead baseline version 3.1 that we used to assign individuals to one of 10 genetic stocks that were initially identified in the Snake River basin by Ackerman et al. (2012). The 10 genetic stocks are the (a) upper Salmon River (UPSALM), (b) Middle Fork Salmon River (MFSALM), (c) South Fork Salmon River (SFSALM), (d) lower Salmon River (LOSALM), (e) upper Clearwater River (UPCLWR), (f) South Fork Clearwater River (SFCLWR), (g) lower Clearwater River (LOCLWR), (h) Imnaha River (IMNAHA), (i) Grande Ronde River (GRROND), and (j) lower Snake River (LSNAKE, Figure 2). These genetic stocks roughly correspond to, or are contained within, major population groups (MPGs) identified in the Snake River basin (Ackerman et al., 2012). This GSI baseline was constructed using *O. mykiss* collected between 1999 and 2013 (Vu et al., 2015), and reflects contemporary steelhead genetic structure in the Snake River basin. Accuracy of this baseline was initially assessed by Vu et al. (2015) using self‐assignment tests in the program gsi_sim (Anderson, 2010; Anderson et al., 2008).

### 2.2 Data collection

We analyzed 31,444 genetic samples collected from putatively natural‐origin adult steelhead at the Lower Granite Dam adult fish trapping facility in spawning run years (July 1st‐June 31st) 2010–2017. A small, nonlethal sample of fin tissue was collected from each fish for genotyping and subsequent GSI analyses. Wild adult steelhead that were not PIT tagged at time of capture in the adult fish facility had one inserted (Ogden, 2019 and references therein). Tissue samples were stored either in 95% nondenatured ethanol or on dry Whatman sampling paper (Lahood, Miller, Apland, & Ford, 2008) prior to extraction. Genomic DNA was extracted using a Nexttec Genomic DNA Isolation Kit for Fish Tissue according to the manufacturer's instructions (www.nexttec.biz), and fish were genotyped at a panel of 180 single nucleotide polymorphisms (SNPs) used in the Columbia River steelhead GSI baseline (Hess, Campbell, et al., 2014). Prior to analysis, we removed locus Omy_IL1b‐163 due to poor performance (Vu et al., 2015). Genotyping was performed using Fluidigm® 96.96 Dynamic Array™ IFCs (chips) for steelhead returning in spawning run years 2010–2015. For spawning run years 2016–2017, genotyping was performed using the Genotyping‐in‐Thousands by sequencing (GT‐seq) protocol (Campbell, Harmon, & Narum, 2015) on an Illumina NextSeq 500 DNA sequencer (Illumina). More detailed methods for, and results from, these genotyping efforts are reported elsewhere (Ackerman et al., 2012, 2014, 2016; Powell et al., 2017; Powell et al., 2018; Vu et al., 2015).

Samples were filtered to include only natural‐origin fish that successfully genotyped at ≥90% of the amplified loci. We identified fish as natural‐origin adult steelhead if they had no marks (e.g., adipose or ventral fin clip), no visible fin erosion (Latremouille, 2003), no coded wire tag (CWT), and did not assign to the Snake River hatchery steelhead parentage based tagging baselines. Samples were then further filtered to include only those adult steelhead that were assigned a spawning location in one of 20 populations described by NMFS (2017, Appendix) based on PIT tag detection (Figure 2). Clear Creek was split from the lower Clearwater River population (CRLMA‐s) due to the fact that previous analyses indicate that steelhead from Clear Creek are genetically more similar to collections in the South Fork Clearwater River population than collections from other drainages in the lower Clearwater River population (Ackerman et al., 2012; Vu et al., 2015). This new population was labeled CRLMA‐s*. Spawning locations were assigned for adults returning in spawning run years 2010–2015 based on the upstream‐most PIT tag detection site (i.e., maximum river kilometer from the mouth of the Columbia River) in a spawn year (Powell et al., 2017). For adults returning in spawning run years 2016–2017, spawning population assignments were determined based on the range of dates across which an individual was present above a given PIT tag detection site (Orme & Kinzer, 2018). A total of 9,613 adult steelhead passed our filtering criteria and were included in the final analysis.

### 2.3 Data analysis

Using PIT detections that reflected presumed natal origin, the corresponding genetic samples were grouped into 20 collections based on populations described in NMFS (2017). We set the minimum population size for this study to be 20 returning PIT tagged adult steelhead (Pruett & Winker, 2008) to minimize bias in population genetic parameter estimates. Populations with greater than 20 individuals sampled within a return year were tested for deviations from Hardy–Weinberg Equilibrium with the R package *HardyWeinberg* version 1.6.3 (Graffelman, 2015; Graffelman & Morales‐Camarena, 2008). We sought to ensure that the final analysis made comparisons across genetically homogenous collections because we sampled returning adults across multiple spawn years. To that end, we tested for genetic differentiation across spawning return years and PIT tag detection sites for all populations with more than 20 detected adult steelhead using 10,000 permutations in the R package *hierfstat* version 0.04‐22 (Goudet & Jombart, 2015). If samples from the same population were statistically differentiated among years, we then tested for differentiation among PIT tag detection sites within years for all years with more than 20 detections. Tests were performed using a Bonferroni adjusted α based on 115 potential simultaneous tests of genetic differentiation (adjusted *α* = 4.35 * 10^–4^). Within populations, only groups of genetically homogenous spawn year and PIT tag detection site combinations were used for analysis.

For GSI assignments, we used the full Expectation‐Maximization algorithm maximum likelihood estimate option in the program gsi_sim (Anderson, 2010; Anderson et al., 2008). Individuals were assigned to one of 10 genetic stocks in the Snake River steelhead baseline version 3.1 (Vu et al., 2015) based on their maximum probability of membership using the allocate sum procedure (Wood, McKinnell, Mulligan, & Fournier, 1987). These genetic stocks roughly correspond to the major population groups (MPGs) into which these 20 populations defined by NMFS (2017) are aggregated. To assess accuracy of individual GSI assignments, we calculated the proportion of fish returning to PIT tag detection sites within the Snake River basin that assigned to the appropriate genetic stock. To quantify the uncertainty of the GSI assignments, we calculated the cumulative distribution of assignment probability observed for each PIT tagged adult steelhead used in the analysis.

We calculated *F*_ST_ using Weir and Cockerham's *θ* (Weir & Cockerham, 1984) in the R package *hierfstat* version 0.04‐22 (Goudet & Jombart, 2015). We averaged pairwise *F*_ST_ values for populations with genetically differentiated spawning run years to have a single *F*_ST_ value for any pair of populations in the analysis. Stream distance was calculated using the river kilometer of the lowest PIT tag detection site within each population reported to PTAGIS (www.ptagis.org).

We examined patterns of IBD using the ratio of $\frac{F_{\text{ST}}}{1‐F_{\text{ST}}}$ as the response variable in a linear regression with stream distance between populations (Rousset, 1997). We tested for an association between stream distance and genetic distance using Mantel tests (Mantel, 1967) with 10,000 permutations.

We used two tests to detect an effect of translocating out‐of‐basin steelhead into the upper Salmon River in the mid‐1960s. First, we tested for a difference between the patterns of IBD including and excluding the upper Salmon River populations (SRLEM‐s, SRPAH‐s, SREFS‐s, SRUMA‐s). We expected that successful steelhead translocations in the mid‐1960s would reduce the overall genetic differentiation (i.e., the slope of the IBD regression) in the Snake River basin. This expected reduction in genetic differentiation is because the stream distance between most steelhead populations in the Snake River basin and Hells Canyon Dam is shorter than between these same populations and the upper Salmon River. We also expected that mimicking the historical association between genetic and geographic distance in the Snake River basin by analytically moving the upper Salmon River populations (SRLEM‐s, SRPAH‐s, SREFS‐s, SRUMA‐s) to Hells Canyon Dam would produce a similar pattern of IBD to the one observed when we removed the upper Salmon River populations from analysis if these translocations were successful. Therefore, our second test examined the difference between the patterns of IBD observed (a) after removing the populations from the upper Salmon River, and (b) after analytically moving these populations to Hells Canyon Dam.

We used a statistical test for comparing the strength of IBD described in Powell (2014) that is analogous to the construction of Mantel based confidence intervals presented in Manly (2007). In this test, if two sets of populations have equivalent patterns of genetic differentiation we expect to observe no relationship between the stream distance matrix of one set of populations (e.g., all populations in their true location) and the residual genetic distance matrix calculated using the slope of the regression line from another set of populations (e.g., populations after removing the upper Salmon River populations). Because the sample set after removing the upper Salmon River populations is used in both steps of the equivalence test, we set this as our reference regression line. If there was a homogenizing effect of translocating steelhead from the mid‐Snake River to the upper Salmon River then we should observe a negative correlation between the residual genetic distance matrix for the full dataset and the stream distance matrix. We would also expect to observe no relationship between the stream distance matrix and the residual genetic distance matrix after moving the upper Salmon River populations to Hells Canyon Dam. We incorporated uncertainty in the estimated slope of the regression line describing IBD after removing the upper Salmon River populations by sampling 10,000 slope coefficients from a Normal distribution with mean equal to the slope of the IBD line and standard deviation equal to the estimated standard error of the slope parameter. For each of these 10,000 sampled slope coefficients we estimated a residual distance matrix and calculated a Mantel test statistic using a randomized stream distance matrix. Significance of IBD relationships were determined based on the proportion of these randomizations that producing a smaller test statistic to that observed with the original stream distance matrix.

We constructed a neighbor‐joining tree for the populations based on Cavalli‐Sforza Edwards chord distance (Cavalli‐Sforza & Edwards, 1967) using PHYLIP v3.5 (Felsenstein, 1993). In addition to PIT tag returns we also included the 2016 broodstocks for Pahsimeroi, Oxbow, and Sawtooth fish hatcheries to provide collections representing the hatchery stocks used to supplement the upper Salmon River. Branch support was estimated by resampling loci 1,000 times, and trees were visualized with Dendroscope version 3.5.9 (Huson & Scornavacca, 2012). Unless otherwise stated all analyses were performed in the R version 3.6.1 (R Core Team, 2019).

## 3 RESULTS

Three loci (Omy_109894‐185, Omy_aldB‐165, OMS00095) were out of Hardy–Weinberg equilibrium due to a deficit of heterozygotes in more than half of the populations analyzed in a given year and were removed from analysis. We observed significant genotypic differentiation across spawning run years and PIT tag detection sites within the lower Clearwater River (CRLMA‐s), Imnaha River (IRMAI‐s), and upper Grande Ronde River (GRUMA‐s) populations (all *p‐values* ≤ .0001). We also observed significant genetic differentiation across PIT tag detection sites within the lower Clearwater River population in 2013 (*p‐value* ≤ .0004) and the Imnaha River population in 2011 (*p‐value* ≤ .0001). Individuals detected on arrays within the lower Clearwater River population in 2013 and the Imnaha River population in 2011 was removed from analysis. A total of 9,159 returning adult steelhead remained in the analysis following the removal of these individuals.

**FIGURE 3:**
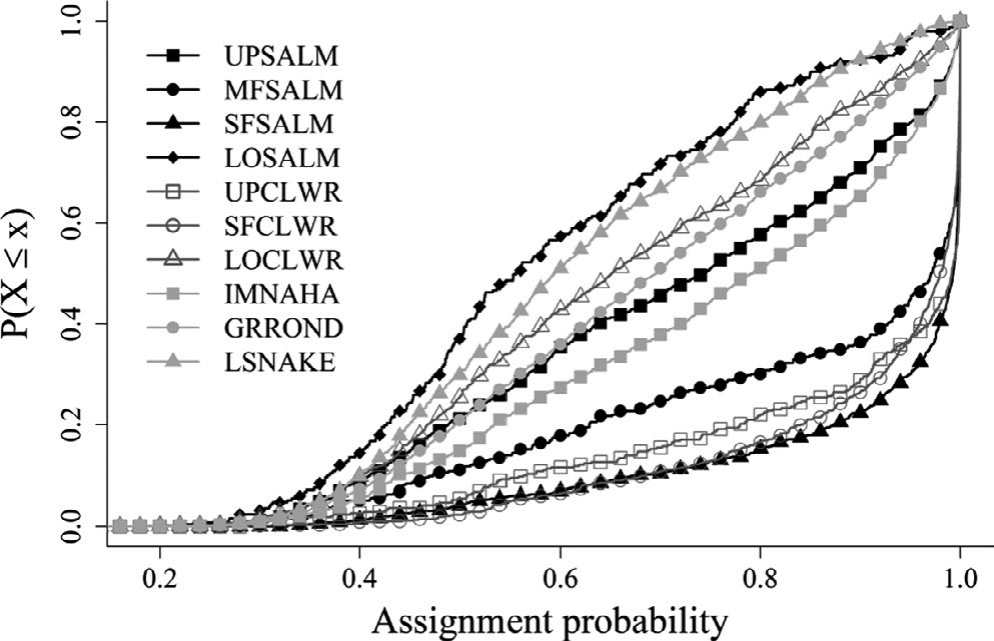
Cumulative distribution functions of assignment probability for the 10 genetic stocks in the Snake River steelhead GSI baseline v3.1. This figure reports the probability that an individual assigned to a given genetic stock (lines) assigns to that genetic stock (*y*‐axis) with at least a specified probability (*x*‐axis)

We observed a general pattern of increasing assignment accuracy of GSI moving upstream from the mouth of the Snake River (Table 1). However, this pattern of increasing assignment accuracy was not directly replicated with a similar pattern of increasing assignment confidence (Figure 3). For example, we observed similar average individual assignment probabilities in the upper Salmon River and the lower Snake River basin genetic stocks (Figure 3).

**TABLE 1:** The proportion of steelhead returning to PIT tag detection sites within a genetic stock that were assigned to one of 10 genetic stocks in the Snake River steelhead GSI baseline version 3.1

	Assigned GSI reporting unit									
	UPSALM	MFSALM	SFSALM	LOSALM	UPCLWR	SFCLWR	LOCLWR	IMNAHA	GRROND	LSNAKE
PIT tag return location										
UPSALM	0.735	0.010	0.000	0.031	0.005	0.006	0.036	0.031	0.081	0.065
MFSALM	0.025	0.887	0.014	0.035	0.004	0.000	0.004	0.011	0.004	0.018
SFSALM	0.010	0.054	0.847	0.026	0.001	0.001	0.012	0.007	0.028	0.015
LOSALM	0.154	0.077	0.038	0.500	0.038	0.038	0.038	0.000	0.038	0.077
UPCLWR	0.000	0.000	0.000	0.000	0.886	0.097	0.014	0.000	0.003	0.000
SFCLWR	0.015	0.000	0.000	0.004	0.100	0.774	0.090	0.001	0.009	0.008
LOCLWR	0.068	0.005	0.005	0.017	0.026	0.028	0.453	0.053	0.176	0.170
IMNAHA	0.077	0.024	0.006	0.037	0.004	0.001	0.048	0.590	0.147	0.065
GRROND	0.079	0.014	0.005	0.027	0.004	0.004	0.103	0.053	0.568	0.144
LSNAKE	0.130	0.014	0.004	0.034	0.006	0.008	0.116	0.039	0.277	0.373

Note

Off diagonal values in each row report the proportion of steelhead that assign to a genetic stock identification reporting unit in which they did not return to spawn.

**FIGURE 4:**
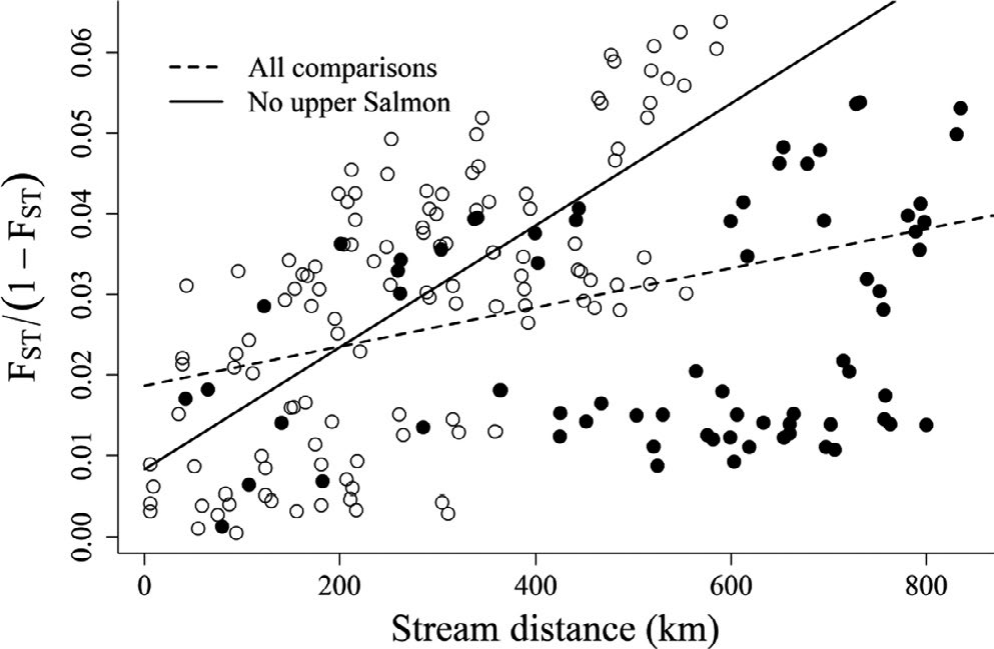
Isolation by distance observed among populations in the Snake River basin calculated with and without populations in the upper Salmon River. Pairwise comparisons including an upper Salmon River population are plotted with filled circles

**FIGURE 5:**
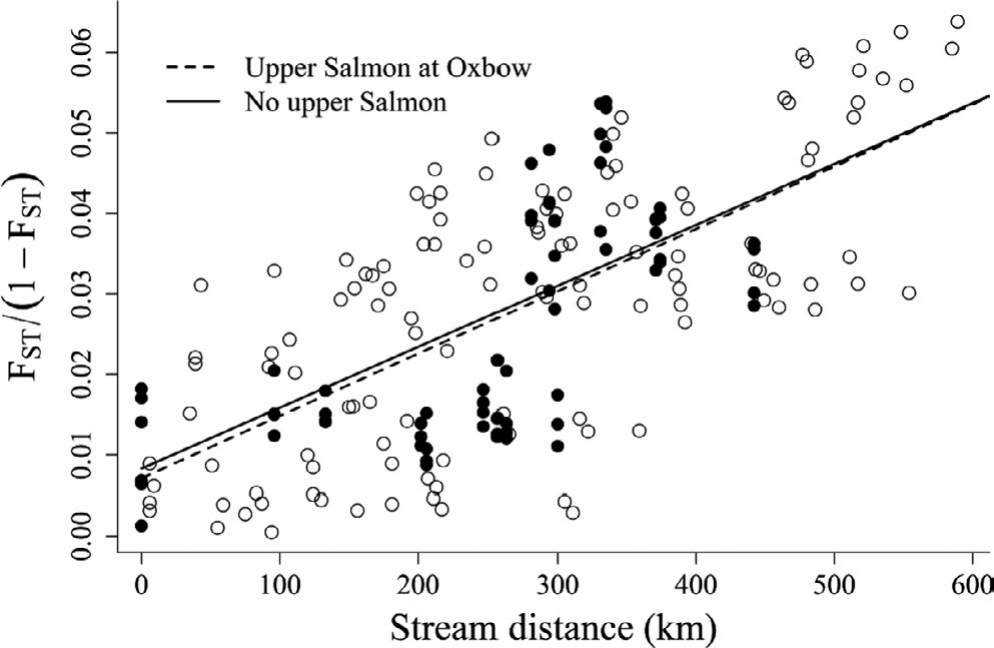
Isolation by distance observed among populations in the Snake River basin after analytically moving upper Salmon River populations to the Oxbow Hatchery trap at the base of Hells Canyon Dam. Pairwise comparisons including an upper Salmon River population are plotted with filled circles

We observed a pattern of IBD in natural‐origin steelhead across the Snake River basin (*p‐value* ≤ .0001, *ρ* = 0.34; Figure 4). We observed a stronger association between genetic distance and geographic distance (*p‐value* = .011) after excluding populations in the upper Salmon River from analysis (*p‐value* ≤ .0001, *ρ* = 0.67; Figure 4). We did not observe a difference in the relationship between genetic distance and geographic distance between the test that excluded populations in the upper Salmon River and the test that analytically moved populations from the upper Salmon River to the Oxbow Hatchery trap at Hells Canyon Dam (*p‐value* = .449; Figure 5).

**FIGURE 6:**
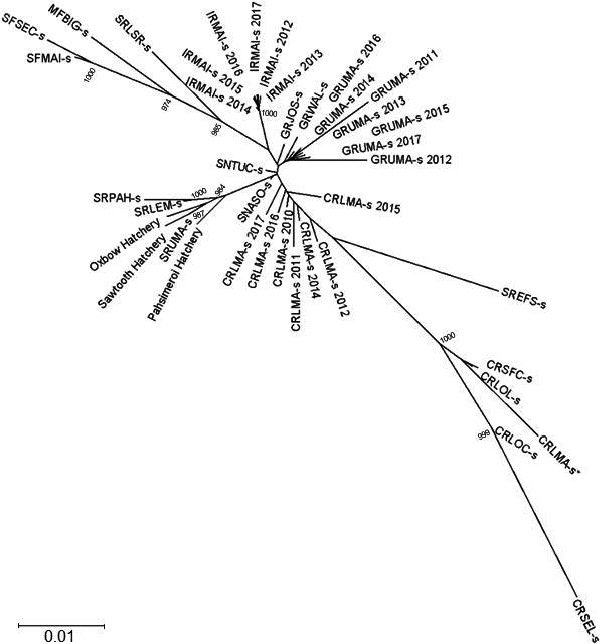
Neighbor‐joining tree based on Cavalli‐Sforza Edwards chord distance for populations of PIT tagged returning adults from SY2010‐2017 and 2016 broodstock collections from the Oxbow, Pahsimeroi and Sawtooth fish hatcheries. Bootstrap support greater than 80% based on 1,000 replicates is reported

Congruent with the results of IBD testing, the phylogeny of Snake River basin steelhead indicated that natural‐origin adult steelhead populations in the upper Salmon River (SRLEM‐s, SRPAH‐s, SRUMA‐s) clustered with the 2016 broodstocks from the Pahsimeroi, Sawtooth, and Oxbow fish hatcheries (Figure 6). The single exception was observed for the samples collected from the East Fork Salmon River (SREFS‐s). The East Fork Salmon River has been stocked with steelhead from the Dworshak Hatchery (N.F. Clearwater), and clusters with populations from the Clearwater River drainage (Figure 6).

## 4 DISCUSSION

We used a comprehensive survey of 9,613 adult steelhead returning to PIT tag detection locations across the Snake River basin to investigate patterns of contemporary genetic structure. We found that populations of wild adult steelhead exhibit a pattern of IBD across the Snake River basin. This finding is consistent with expectations based on work performed across the eastern Pacific in both coastal (Arciniega et al., 2016; Garza et al., 2014; Heath, Pollard, & Herbinger, 2001; Pearse, Donohoe, & Garza, 2007; Pearse et al., 2011) and interior lineages (Matala et al., 2014). However, the strength of the association between geographic and genetic distance in the Snake River basin has likely been reduced as a result of historic supplementation in the upper Salmon River using steelhead from the Snake River upstream of Hells Canyon Dam (Figure 5). Similar patterns of reduced contemporary genetic divergence were also observed in coastal California watersheds when IBD among museum collections was compared to contemporary conditions after hatchery supplementation (Pearse et al., 2011).

Although hatchery production and supplementation across the West Coast of the United States of America is ubiquitous, it has been driven by different conservation and management objectives. The reasons for hatchery production in the upper Salmon River have been twofold: (a) to compensate for the loss of anadromous steelhead populations in the middle and upper Snake River following the construction of Hells Canyon Dam, and (b) to provide hatchery steelhead to meet sport and tribal harvest mitigation goals in the upper Salmon River. To this end, Pahsimeroi Fish Hatchery was founded using steelhead trapped at Hells Canyon Dam (Reingold, 1967) and Sawtooth Fish Hatchery was founded using smolts from Pahsimeroi Fish Hatchery (Moore, 1983). While hatchery production efforts in the upper Salmon River have also included the release of juvenile steelhead from the Clearwater River basins (Stiefel, 2013), the results of the genetic analyses described in this paper and by previous authors (Blankenship et al., 2011; Nielsen, Byrne, Graziano, & Kozfkay, 2009) generally did not detect a large influence of these releases on the population genetic structure in the basin. For example, our phylogenetic analysis was consistent with previous research in the Snake River basin that indicated that current natural‐origin steelhead populations in the upper Salmon River share a more recent common ancestor with steelhead from the Oxbow Fish Hatchery (and other middle Snake River populations, Blankenship et al., 2011; Nielsen et al., 2009). However, we observed the influence of past releases of steelhead from Dworshak Fish Hatchery in the East Fork Salmon River (for a summary of hatchery releases see pg. 164 in ICTRT, 2003), with this population clustering with collections from the Clearwater River basin in our phylogeny.

The contemporary genetic structure helps explain the observed patterns in GSI analyses in the Snake River basin. The observed patterns of IBD indicate that despite the populations in the upper Salmon River being the farthest from the mouth of the Snake River, these populations are genetically more similar to steelhead populations lower in the Snake River basin. However, a higher proportion of the fish arriving at PIT tag detection locations in the upper Salmon River is assigned to this genetic stock than other lower basin genetic stocks (Table 1) despite similar patterns of membership probability for individuals in the upper Salmon River and genetic stocks in the lower basin (Figure 3). The disparate patterns of assignment confidence and accuracy evident in the upper Salmon River may be explained by a disconnection between physical distance and genetic distance. Because the per generation increase of *F*_ST_ is dependent on the migration rate and the reciprocal of twice the effective population size (Hartl & Clark, 2007) the increase in genetic divergence among populations is a slow process. Therefore, our confidence in genetic stock assignments in the upper Salmon River is reduced as a result of the lower genetic divergence within the Snake River basin due to the success of past translocation efforts, while the accuracy of these assignments reflects a low level of straying due to their geographic isolation.

Genetic stock identification has been an important tool for monitoring wild steelhead in the Snake River Evolutionarily Significant Unit despite the low genetic differentiation among steelhead populations from the upper Salmon River and middle Snake Rivers. The primary use of GSI in the Snake River basin has been to parse the total wild escapement of adults that pass Lower Granite Dam for annual stock abundance estimation (Camacho et al., 2019). Powell et al. (2018) showed that estimated genetic stock proportions are unbiased and that individual assignment accuracy for the Middle Fork Salmon River, South Fork Salmon River, and Upper Clearwater River reporting groups is high (>90%). These watersheds are solely managed for wild fish production with no history of hatchery supplementation, characteristics that make them priorities for monitoring and conservation. However, prior to the implementation of GSI, abundance estimates for these areas were largely unavailable (Busby et al., 1996; Good, Waples, & Adams, 2005) due to the location of many populations in remote or wilderness areas, and environmental conditions at the time of spawning preventing the use of traditional counting methodologies (weirs, rotary screw traps, and redd count surveys). Therefore, GSI remains a critical tool for monitoring wild steelhead in the Snake River basin because of how difficult it is to estimate abundance in these populations with other methods.

Although the results presented here (and previously) indicate some limitations of differentiating stocks that have shared ancestries from translocation and supplementation efforts, there are opportunities to increase assignment accuracy by incorporating SNPs under selection (Ackerman, Habicht, & Seeb, 2011) and by moving to loci that contain multiple alleles (i.e., microhaplotypes) over single‐SNP loci (Baetscher, Clemento, Ng, Anderson, & Garza, 2018). Advances in reduced‐representation sequencing and whole‐genome sequencing make finding these loci much more cost‐effective and efficient than previous methods (Andrews, Good, Miller, Luikart, & Hohenlohe, 2016; Li & Wang, 2017) and will be the focus of our GSI work moving forward.

## CONFLICT OF INTEREST

The authors report no conflict of interest.

## AUTHOR CONTRIBUTIONS

**John H. Powell:** Conceptualization (equal); formal analysis (lead); visualization (lead); writing – original draft (lead); writing – review & editing (supporting). **Matthew R. Campbell:** Conceptualization (equal); funding acquisition (lead); project administration (lead); resources (lead); supervision (lead); writing – original draft (supporting); writing – review & editing (lead).

## Data Availability

Individual genotypes and return location are archived on Dryad (https://doi.org/10.5061/dryad.98sf7m0gj).

## Appendix 1

Steelhead populations and PIT tag detection locations in the Snake River basin. The alphanumeric site code, site description, and location are shown. Reproduced from Powell et al. (2018).

**Table:**

Site code	Site description	Latitude	Longitude
SRUMA‐s – Salmon River upper mainstem			
STL	Sawtooth Hatchery	44.15317	−114.88347
SAWT	Sawtooth Hatchery	44.15068	−114.88366
VC1	Valley Creek, lower site	44.22178	−114.93154
VC2	Valley Creek, upper site	44.21860	−114.94216
YFK	Yankee Fork Salmon River	44.28761	−114.72075
CEY	Cearley Creek Side Channel	44.33906	−114.72164
SREFS‐s—East Fork Salmon River			
SALEFT	East Fork Salmon River	44.12878	−114.41867
SALREF	East Fork Salmon River	44.12878	−114.41867
SRPAH‐s—Pahsimeroi River			
PAHH	Pahsimeroi Hatchery	44.68414	−114.03947
PAHTRP	Pahsimeroi Hatchery Trap	44.68453	−114.04044
SRLEM‐s—Lemhi River			
LLR	Lower Lemhi River	45.17635	−113.88512
AGC	Agency Creek, Lemhi River basin	44.95674	−113.63954
LRW	Lemhi River Weir	44.86612	−113.62475
HYC	Hayden Creek	44.86159	−113.63215
WPC	Wimpey Creek, Lemhi River basin	45.09794	−113.72050
BHC	Bohannon Creek	45.11777	−113.74130
LBS	Big Springs Creek	44.72735	−113.43321
CRC	Carmen Creek, Salmon River basin	45.24648	−113.89347
KEN	Kenny Creek in‐stream arrays	45.02703	−113.65801
WIMPYC	Wimpey Creek, Lemhi River basin	45.13571	−113.66249
LLS	Lemhi Little Springs instream	44.77865	−113.54208
BTC	Big Timber Creek	44.68811	−113.37041
CAC	Canyon Creek ISA at km 1	44.69201	−113.35471
HAYDNC	Hatden Creek, Lemhi River basin	44.75277	−113.71298
CARMEC	Carmen Creek – tributary to the Salmon River	45.30850	−113.80428
MFBIG‐s—Big, Camas and Loon Creeks			
TAY	Big Creek	45.10387	−114.84970
BIG2C	Big Creek, Middle Fork Salmon River	45.15776	−115.12014
SFMAI‐s—South Fork Salmon River			
KRS	South Fork Salmon River, Krassel	44.97840	−115.72700
ESS	East Fork South Fork Salmon River	44.95621	−115.53315
KNOXB	Knox Bridge, South Fork Salmon River	44.65552	−115.70240
JOHNSC	Johnson Creek	44.73393	−115.54860
SFSEC‐s—Secesh River			
ZEN	Secesh River near Zena Creek Ranch	45.03330	−115.73302
SRLSR‐s—Little Salmon and Rapid Rivers			
RAPH	Rapid River Hatchery	45.35368	−116.39458
RPDTRP	Rapid River smolt trap	45.35841	−116.38804
CRLOC‐s—Lochsa River			
FISTRP	Fish Creek Trap	46.34011	−115.35513
LRL	Lower Lochsa River	46.14573	−115.59650
CRSEL‐s—Selway River			
SW1	Lower Selway River	46.11032	−115.56589
CRSFC‐s—South Fork Clearwater River			
SC1	South Fork Clearwater, rkm 1	46.13685	−115.98091
SC2	South Fork Clearwater, rkm 2	46.12749	−115.97730
CROTRP	Crooked River Trap	45.82120	−115.52775
CLWRSF	South Fork Clearwater River	45.82815	−115.95471
CROOKR	Crooked River	45.76266	−115.54144
RRT	Red River satellite facility	45.71118	−115.34715
CRLOL‐s—Lolo Creek			
LC1	Lolo Creek, rkm 21	46.29443	−115.97612
LC2	Lolo Creek, rkm 25	46.29056	−115.93415
CRLMA‐s*—Clearwater lower mainstem in South Fork Clearwater River GSI genetic stock			
CLC	Clear Creek near Kooskia National Fish Hatchery	46.13274	−115.95018
KOOS	Kooskia National Fish Hatchery	46.12971	−115.94683
CRLMA‐s—Clearwater lower mainstem			
PCM	Pine Creek, Potlatch River	46.63067	−116.59684
POTREF	East Fork Potlatch River	46.84772	−116.34912
POTRWF	West Fork Potlatch River	46.92386	−116.45156
KHS	Big Bear Creek	46.61912	−116.64685
LBEARC	Little Bear Creek, Potlatch River watershed	46.67401	−116.70727
BIGBEC	Big Bear Creek, Potlatch River	46.73001	−116.62114
LAP	Lapwai Creek	46.44327	−116.81254
SWT	Sweetwater Creek	46.36979	−116.79508
WEB	Webb Creek	46.32599	−116.83197
MIS	Mission Creek	46.36706	−116.73560
JUL	Potlatch River near Juliaetta	46.56532	−116.70932
HLM	Potlatch River near Helmer	46.79901	−116.42841
IRMAI‐s—Imnaha River			
COC	Cow Creek	45.76774	−116.74404
IR1	Imnaha River, rkm 7	45.76112	−116.75066
IR2	Imnaha River, rkm 10	45.74284	−116.76456
BSC	Big Sheep Creek	45.50649	−116.85067
CMP	Camp Creek at rkm 2—Imnaha River	45.55182	−116.86694
LSHEEF	Little Sheep Facility	45.47782	−116.93025
IR3	Imnaha River, rkm 41	45.49004	−116.80393
CZY	Crazyman Creek at 0.6 km	45.22930	−116.84478
FREEZC	Freezeout Creek—tributary to Imnaha River	45.35041	−116.76217
GUMBTC	Gumboot Creek, Imnaha River basin	45.15572	−116.94111
IR4	Imnaha Weir downstream array	45.19446	−116.86877
IR5	Imnaha Weir upstream array	45.19319	−116.86859
HORS3C	Horse Creek, Imnaha River	45.54951	−116.72727
IMNAHW	Imnaha River Weir	45.19428	−116.86866
MAHOGC	Mahogany Creek, Imnaha River	45.20021	−116.89999
GRUMA‐s—Grande Ronde upper mainstem			
CATHEW	Catherine Creek Weir	45.19096	−117.82862
CCW	Catherine Creek Ladder/Weir	45.19096	−117.82862
UGR	Upper Grande Ronde River	45.59334	−117.90312
LOOH	Lookingglass Hatchery	45.73154	−117.86441
LOOKGC	Lookingglass Creek	45.75720	−117.96001
CATHEC	Catherine Creek	45.20967	−117.88785
GRANDW	Grande Ronde River weir	45.24896	−118.38898
GRWAL‐s—Wallowa River			
WR1	Wallowa River at river km 14	45.63368	−117.73376
WALH	Wallowa Hatchery	45.41757	−117.30157
LOSTIW	Lostine River Weir	45.54327	−117.48450
BCANF	Big Canyon Facility	45.61904	−117.69863
WALLOR	Wallowa River	45.54922	−117.45906
MINAMR	Minam River	45.33888	−117.60018
GRJOS‐s—Joseph Creek			
JOC	Joseph Creek	46.03002	−117.01604
JOSEPC	Joseph Creek, Grande Ronde River basin	45.89979	−117.20915
SNASO‐s—Asotin Creek			
ACM	Asotin Creek, Mouth	46.34137	−117.05571
GEORGC	George Creek, Asotin Creek watershed	46.19230	−117.19884
ASOTIC	Asotin Creek, Snake River above Clarkston, WA	46.33064	−117.18195
ACB	Asotin Creek, Cloverland	46.32545	−117.10852
AFC	Asotin Creek, North Fork/South Fork Junction	46.27230	−117.29243
CCA	Charley Creek	46.28846	−117.28250
ALPOWC	Alpowa Creek, lower Snake River	46.40235	−117.39827
ALMOTC	Almota Creek—tributary to Snake River	46.70161	−117.35935
PENAWC	Penawawa Creek—tributary to Snake River	46.74777	−117.54136
TENMC2	Tenmile Creek—tributary to Snake River	46.19525	−117.04185
CHARLC	Charley Creek—Asotin Creek watershed	46.27241	−117.43662
SNTUC‐s—Tucannon River			
LTR	Lower Tucannon River	46.54419	−118.16290
MTR	Middle Tucannon River	46.50526	−118.01628
UTR	Upper Tucannon River	46.41584	−117.73832
TFH	Tucannon Hatchery	46.30965	−117.65715
TUCR	Tucannon River	46.39572	−117.71120
TUCH	Tucannon River Hatchery	46.32011	−117.66284
PATAHC	Patah Creek—tributary to Tucannon River	46.47285	−117.54508
